# Supplementary material for: From sequence to enzyme mechanism using multi-label machine learning
Source: BMC Bioinformatics. 2014 May 19;15:150. doi: 10.1186/1471-2105-15-150 (PMC4229970; doi:10.1186/1471-2105-15-150)
Supplement: Additional file 2 — Java code of ml2db. Additional file ml2db_code.tar.gz contains the Java source code to run the multi-label machine learning experiments and save the results to database. The code’s Javadoc is included. [file 1471-2105-15-150-S2.zip › additional file 2/ml2db/utils/doc/edu/cornell/lassp/houle/RngPack/Ranecu.html]

Ranecu


JavaScript is disabled on your browser.


- Overview
- Package
- Class
- Use
- Tree
- Deprecated
- Index
- Help

- Prev Class
- Next Class

- Frames
- No Frames

- All Classes

- Summary:
- Nested |
- Field |
- Constr |
- Method

- Detail:
- Field |
- Constr |
- Method


edu.cornell.lassp.houle.RngPack

## Class Ranecu

- java.lang.Object
- - cern.colt.PersistentObject
  - - edu.cornell.lassp.houle.RngPack.RandomElement
    - - edu.cornell.lassp.houle.RngPack.RandomSeedable
      - - edu.cornell.lassp.houle.RngPack.Ranecu

- All Implemented Interfaces:
  :   java.io.Serializable, java.lang.Cloneable

  ---

    

  ```
  public class Ranecu
  extends RandomSeedable
  ```

  Ranecu is an advanced multiplicative linear congruential random number
  generator with a period of aproximately 10^18^. Ranecu is a direct
  translation from Fortran of the **RANECU** subroutine published in the
  paper   
  F. James, Comp. Phys. Comm. **60** (1990) p 329-344   
  The algorithm was originally described in   
  P. L'Ecuyer, Commun. ACM. **1988** (1988) p 742   

  More info .

  Source code  is available.

  Version:
  :   1.0

  Author:
  :   Paul Houle  (E-mail:
      houle@msc.cornell.edu)

  See Also:
  :   Serialized Form

- - ### Field Summary

    Fields

    | Modifier and Type | Field and Description |
    | --- | --- |
    | `static int` | `DEFSEED1` default iseed1 = 12345 |
    | `static int` | `DEFSEED2` default iseed2 = 67890 |
  - ### Constructor Summary

    Constructors

    Constructor and Description

    `Ranecu()`

    Initialize RANECU with the default seeds from James.

    `Ranecu(java.util.Date d)`

    `Ranecu(int s1,
    int s2)`

    Initialize RANECU with two specified integer seeds.

    `Ranecu(long l)`
  - ### Method Summary

    Methods

    | Modifier and Type | Method and Description |
    | --- | --- |
    | `long` | `getSeed()` |
    | `double` | `raw()` Returns a uniformly distributed pseudo-random number. |
    | `void` | `raw(double[] d, int n)` This is an inline version that returns an array of doubles for speed. |

    - ### Methods inherited from class edu.cornell.lassp.houle.RngPack.RandomSeedable

      `ClockSeed, ClockSeed`
    - ### Methods inherited from class edu.cornell.lassp.houle.RngPack.RandomElement

      `choose, choose, gaussian, gaussian, powlaw, raw, uniform`
    - ### Methods inherited from class cern.colt.PersistentObject

      `clone`
    - ### Methods inherited from class java.lang.Object

      `equals, getClass, hashCode, notify, notifyAll, toString, wait, wait, wait`

- - ### Field Detail


    - #### DEFSEED1

      ```
      public static int DEFSEED1
      ```

      default iseed1 = 12345


    - #### DEFSEED2

      ```
      public static int DEFSEED2
      ```

      default iseed2 = 67890
  - ### Constructor Detail


    - #### Ranecu

      ```
      public Ranecu()
      ```

      Initialize RANECU with the default seeds from James.


    - #### Ranecu

      ```
      public Ranecu(java.util.Date d)
      ```


    - #### Ranecu

      ```
      public Ranecu(int s1,
            int s2)
      ```

      Initialize RANECU with two specified integer seeds. Use this
      to introduce repeatable seeds. Equivalent to
      `Ranecu(s1*(long) Integer.MAX_VALUE)+s2)`

      Parameters:
      :   `s1` - seed integer 1 (MSW)
      :   `s2` - seed integer 2 (LSW)


    - #### Ranecu

      ```
      public Ranecu(long l)
      ```
  - ### Method Detail


    - #### getSeed

      ```
      public long getSeed()
      ```

      Returns:
      :   the current generator state as a long. Can be used to restart the
          generator where one left off.


    - #### raw

      ```
      public final double raw()
      ```

      **Description copied from class: `RandomElement`**

      Returns a uniformly distributed pseudo-random number. The abstract method
      that must be defined to make a working RandomElement. See the class
      `RandomJava` for an example of how to do this.

      **Specified by:**
      :   `raw` in class `RandomElement`

      Returns:
      :   a uniformly distributed pseudo-random double in the range (0,1).

      See Also:
      :   `RandomElement.raw()`


    - #### raw

      ```
      public final void raw(double[] d,
             int n)
      ```

      This is an inline version that returns an array of doubles for speed.

      **Overrides:**
      :   `raw` in class `RandomElement`

      Parameters:
      :   `d` - array to be filled with doubles
      :   `n` - number of doubles to generate


- Overview
- Package
- Class
- Use
- Tree
- Deprecated
- Index
- Help

- Prev Class
- Next Class

- Frames
- No Frames

- All Classes

- Summary:
- Nested |
- Field |
- Constr |
- Method

- Detail:
- Field |
- Constr |
- Method
